# Chemopreventive Activity of Honokiol against 7, 12 - Dimethylbenz[a]anthracene-Induced Mammary Cancer in Female Sprague Dawley Rats

**DOI:** 10.3389/fphar.2017.00320

**Published:** 2017-05-31

**Authors:** Zhenyu Wang, Xingyi Zhang

**Affiliations:** ^1^Department of Breast Surgery, The Second Hospital of Jilin UniversityChangchun, China; ^2^Department of Thoracic Surgery, The Second Hospital of Jilin UniversityChangchun, China

**Keywords:** honokiol, chemoprevention, DMBA, breast cancer

## Abstract

Breast cancer is a predominant cause of death in women across the globe. Chemoprevention by using natural, dietary or synthetic products has been appearing to be a fascinating approach to combat the growing burden of breast cancer. In the current study, we intended to explore the mechanisms of chemopreventive action of honokiol against 7, 12 - dimethylbenz[a]anthracene (DMBA)-induced mammary cancer in female Sprague Dawlely (SD) rats. We induced mammary cancer in SD rats by administering single dose of DMBA (80 mg/kg) through intra gastric route. Chemopreventive effects of honokiol (80 mg/kg, i.p.) were confirmed from its ameliorating effect on the DMBA-induced anomalies such as liver marker enzymes, Phases I and II metabolizing enzymes and oxidative stress markers. Further, honokiol reversed the DMBA-induced abnormalities in inflammatory cytokines levels and serum tumor markers. Additionally, histopathological examination of mammary tissue and protein expression analysis of NF-κB revealed that honokiol is effective against DMBA-induced mammary cancer. In summary, the results of our study support the chemopreventive feature of honokiol in mammary cancer.

## Introduction

Breast cancer is a complicated ailment that can precipitate due to anomalies in several physiological cell signaling pathways. Presently, breast cancer is an alarming global public health problem, as it is a key cause of illness and death in women. In fact, in females, it is the second most reason for cancer-induced deaths. Furthermore, in the year 2016, breast cancer alone is anticipated to reach 29% all newly diagnosed cancers in women (Siegel et al., 2016). The National Central Cancer Registry of China reported that in China, after lung cancer breast cancer is the principal reason of cancer-related deaths in women younger than 45 years (Chen et al., 2016). There are various risk factors, which could be ascribed to breast cancer; among them predominant factors are genetic vulnerability (mutations in BRCA 1 and BRCA 2), exposure of radiation, overweight and obesity, and alcohol addiction. Furthermore, breast cancer is also associated with age and estrogen exposure. A steroidal hormone, principally estrogen, can cause the induction and growth of breast cancer (Bishayee et al., 2013). Presently, there are 2 SERMs (selective estrogen receptor modulators) namely tamoxifen and raloxifene which are approved by the United States FDA for the chemoprevention of breast cancer in women who are highly susceptible. However, concerns exist over the potential adverse effects, thus limiting the extensive and chronic use of these drugs. Furthermore, the effects of these drugs may not completely eliminate the chances of getting breast cancer. Therefore, there is a scope in this indication for newer interventions (McDonald et al., 2016).

Several clinical investigations have been executed for effectively combating breast cancer. Eventually, there has been a sporadic improvement in the breast cancer management thru the last few decades. Clinical studies demonstrate that early diagnosis and amplified use of hormonal and adjuvant chemotherapies decrease the breast cancer associated illness and deaths. However, applicability of all these modalities is mostly limited by their inordinate toxicity, inadequate efficacy, therapeutic resistance and treatment-related morbidity. Thus, further effective modalities are definitely required to fight against diseases and deaths associated with breast cancer (McDonald et al., 2016).

In the recent past, cancer chemoprevention has been in the vogue in a substantial number of clinical and preclinical studies of several cancer types. Indeed, the United States Preventive Services Task Force (USPSTF) and the American Society of Clinical Oncology (ASCO) endorsed breast cancer chemoprevention in their recent guidelines (Alés-Martínez et al., 2015). Cancer chemoprevention involves utilizing natural, dietary or synthetic products to suppress or prevents cancer. Interesting findings from the several studies made cancer chemoprevention as a potential approach to fight cancer (Bozovic-Spasojevic et al., 2012; Kasala et al., 2016). An appreciable amount of natural substances, consisting of phytochemicals and dietary substances have been found to attenuate breast cancer via affecting cell proliferation, cell differentiation, angiogenesis, apoptosis and a few other cellular transduction pathways (Vadodkar et al., 2012). Indeed, probable application of several dietary supplements and natural substances in preventing breast cancer evaluated by clinical studies too (Kado et al., 2012).

**FIGURE 1:**
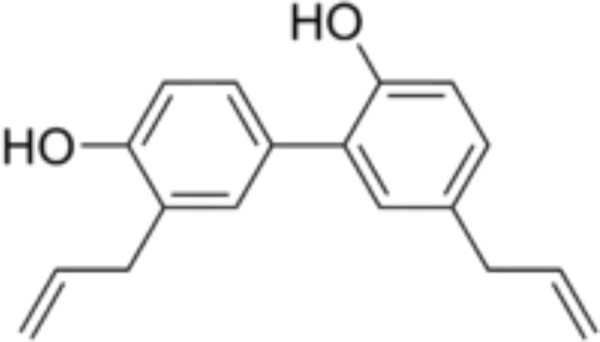
Chemical structure of honokiol. [2-(4-hydroxy-3-prop-2-enyl- phenyl)-4-prop-2-enyl-phenol]. (CAS number: 35354-74-6; molecular formula: C_18_H_18_O_2_; molecular weight: 266.33 g/mol).

The ancient medicine practices in China have had employed the root and stem bark of Magnolia plants for treating several ailments such as anxiety and gastrointestinal ailments (Fried and Arbiser, 2009). Therapeutic effects of Magnolia species have been ascribed to honokiol, a natural phenolic substance present in its extracts (Fried and Arbiser, 2009) (**Figure 1**). Honokiol demonstrated to have several activities such as antibacterial, anti-thrombocytic, anti-inflammatory and anxiolytic effects (Fried and Arbiser, 2009). Furthermore, anticancer effect of honokiol has been evaluated in several *in vitro* and *in vivo* studies (Arora et al., 2012). Honokiol acts through regulating several cellular pathways and regulate cellular targets, which can affect apoptosis, cell differentiation, cell growth and survival of cancer cells. Previous experimental investigations reported that honokiol shows cancer cell growth arrest and induce cell death by cell-line/tumor-type specific signaling pathways. Indeed, honokiol affects multiple targets such as nuclear factor kappa B (NF-κB), STAT3, and EGFR that show excessive significance on cancer initiation and development (Arora et al., 2012).

The site specific cancer-causing agent 7, 12-dimethylbenz[a]anthracene (DMBA) is generally used to evoke mammary carcinoma in Sprague Dawley (SD) rats. DMBA could either be employed as an initiator or promoter for inducing mutations in genes, responsible for breast cancer (Arora et al., 2012). In the current study, we tested the chemopreventive potential of honokiol in DMBA-evoked mammary cancer in female SD rats.

## Materials and Methods

### Chemicals

7, 12 - dimethylbenz[a]anthracene and honokiol were obtained from TCI chemicals, China. SOD, catalase, IL-1β, IL-6 and TNF-α kit were bought from Sigma–Aldrich, United States. All other chemicals employed in the study were of analytical grade.

### Animals

Adult female Sprague Dawley rats of 160 ± 20 g body weight, (6–8 weeks age) were used in the present study. Animals were procured from the central animal laboratory of our institution and were kept on acclimatization for about 2 weeks. Animals were fed with pellet diet and water *ad libitum* throughout the investigational tenure. Standard laboratory conditions were maintained under regulated atmosphere (12:12 h light/dark cycles with an ambient temperature of 22 ± 3°C and humidity at 50 ± 10%.

#### Ethics Statement

All animal experiments were carried out strictly in accordance with International Ethical guidelines and the National Institutes of Health Guide concerning the Care and Use of Laboratory Animals. The experiments were approved by the Institutional Animal Care and Use Committee of The Second Hospital of Jilin University.

### Study Plan

#### Tumor Induction

The 80 mg/kg of DMBA was administered to SD rats for tumor induction, as this dose is sufficient to cause 100% tumor incidence (Fackenthal and Olopade, 2007). DMBA takes 8–10 weeks approximately to induce tumors in female SD rats. Animals were sacrificed at the completion of the study period, i.e., after 16 weeks, blood and mammary tissues were collected for further analysis (Al-Saeedi, 2014).

#### Experimental Design

Animals were randomized into four different groups consisting of six animals in each. The groups termed as number I, II, III, and IV.

• Group I (Vehicle control): Rats were administered sesame oil thrice a week for 18 weeks via intragastric route.
• Group II (Drug control): Honokiol (80 mg/kg, i.p.) alone treated (thrice a week for 18 weeks) animals; served as drug control group.
• Group III (DMBA control): Animals were administered with single dose of 0.5 ml DMBA (80 mg/kg) in sesame oil during week ‘0,’ and then rats were given only sesame oil during rest of the study period (1–16 weeks).
• Group IV (Pre-treatment): Animals were subjected to pre-treatment with honokiol (80 mg/kg, i.p.) from -2 to 0 weeks and 1–16 weekly thrice, and DMBA was administered during the week ‘0.’

**FIGURE 2:**
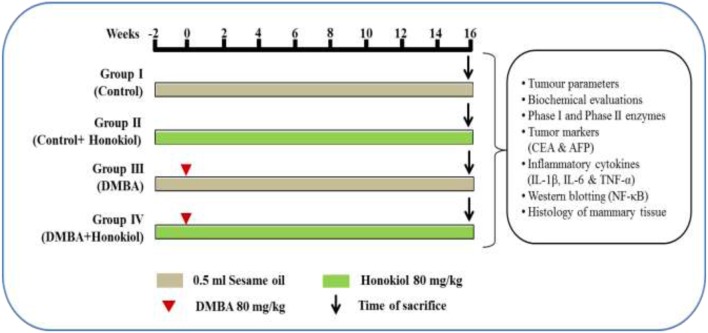
Study plan and treatment schedule. Animals were assigned into four different groups each comprising of six each. The groups were named as Groups I, II, III, and IV. The Group I is termed as ‘vehicle control’ and administered sesame oil from the –2 to 16th week via intragastric route. Group II is termed as ‘drug control’ and administered honokiol alone thrice a week during entire study tenure. Group III is named as ‘DMBA control’ and tumors were induced in the ‘0’th week by a single dose of 0.5 ml DMBA (80 mg/kg) in sesame oil, and the rats were given only sesame oil during entire study period (–2 to 0 and 1–16 weeks). Group IV termed as pre-treatment and subjected to pre-treatment with honokiol (80 mg/kg) i.p. at –2 to 0 and 1–16 weekly thrice, and DMBA was administered in the ‘0’th week. After 16 weeks post-DMBA exposure, all animals were sacrificed. Finally, part of tumors were fixed in 10% buffered formalin for histopathological analysis and remaining part of tumors were processed for molecular and biochemical analysis. Similarly, blood was collected for the biochemical analyses.

All animals were sacrificed 16 weeks after post DMBA. Finally, a part of tumor was fixed in formalin for histopathological analysis and remaining parts of tumor were processed for molecular and biochemical analysis. Similarly, blood was also collected for the analysis of various biochemical parameters (Detailed experimental protocol illustrated in the **Figure 2**).

### Quantification of Body and Tumor Parameters

The body weight of the experimental rats was measured periodically during the experimental tenure. The tumor volume was measured using, Π/6 × (a) 2/(b), where ‘a’ is the shortest and ‘b’ is the longest length of the tumor. Tumor number is the over-all number of tumors developed and tumor incidence is the fraction of tumors developed in each animal group (Shibata et al., 2010).

### Estimation of Liver Marker Enzymes

The liver function marker enzymes such as aspartate aminotransferase (AST) and alanine aminotransferase (ALT) were estimated by the procedure described by Bergmeyer et al. (1978). Alkaline phosphatase and acid phosphatases were quantified as described in a previous method, by employing disodium phenyl phosphate as the substrate (Bergmeyer et al., 1978; Anbuselvam et al., 2007). Lactate dehydrogenase (LDH) was estimated by the King method. Gamma-glutamyl transpeptidase (GGT) was quantified using Rosalki and Rau protocol (Rosalki and Rau, 1972).

### Quantification of Phase I and II Enzymes

The mammary tissues were separated aseptically from the animals and then cleaned with ice-cold sterile 1.15% KCl and homogenized. The resultant homogenates were centrifuged to get the S9 fraction of microsomes, which was used for further analysis. The Phase I detoxification enzymes (Cytochrome P450 and Cytochrome b5) were quantified by the Omura and Sato method (OMURA and SATO, 1964). The ethoxyresorufin O-decarboxylase (EROD) and pentoxyresorufin O-decarboxylase (PROD) activities were quantified by the formation of resorufin spectrofluorimetrically at excitation and emission wavelength of 536 and 585 nm, respectively (Burke and Mayer, 1974). Similarly, the Phase II detoxification enzymes [glutathione *S*-transferase (GST) and quinone reductase (QR)] were measured by previously described methods (Habig and Jakoby, 1981).

### Assay for Oxidative Stress Parameters

The enzymatic antioxidant catalase was measured by means of commercially available catalase assay kit, following the instructions provided by the manufacturer (Sigma–Aldrich Corp., St. Louis, United States). Mammary tissue was separated from the animals and homogenized in phosphate buffer saline (pH 7.4, 8% w/v). Supernatants were taken for the CAT assay after centrifugation of homogenates at 15,000 *g* for 20 min at 4°C. Absorbance was read at 520 nm and the results were exhibited as mMol/min/mg of protein. Similarly, SOD was quantified by using SOD assay kit (Sigma–Aldrich, St. Louis, MO, United States). Absorbance was read at 440 nm on microplate reader and results were demonstrated as U/mg of protein. MDA and nitrate levels were also estimated by using commercially available kits.

The non-enzymatic antioxidants [Vitamin C, Vitamin E and reduced glutathione (GSH)] were also determined. In the GSH assay, 1 ml of the sample was precipitated with 1 ml of TCA and centrifuged at 1200 *g* for 20 min. To 0.5 ml of supernatant, 2 ml of 5, 50-dithiobis-(2-nitrobenzoic acid) (DTNB) was added and the color developed was read immediately at 412 nm using a spectrophotometer (Moron et al., 1979). Vitamin E assay determined the levels of ferric ions which were reduced to ferrous ions in the presence of tocopherol and bathophenanthroline to form a pink colored substance, which was read at 520 nm using a spectrophotometer (Desai, 1984). In the Vitamin C assay, 1 ml of ethanol was added to 1 ml of sample and then mixed thoroughly after which 3 ml of petroleum ether was added and the reaction mixture was centrifuged. The supernatant was evaporated to dryness and 0.2 ml each of bathophenanthroline, ferric chloride and O-phosphoric acid were added to reach a total volume of 3 ml with ethanol. The color developed was measured at 530 nm (Omaye et al., 1979).

### Enzyme Linked Immune Sorbent Assay for Proinflammatory Cytokines

Quantification of TNF-α, IL-1β, and IL-6 was carried out by ELISA. Briefly, 10% tissue homogenate was obtained from the mammary tissue samples in PBS consisting of 1% protease inhibitor cocktail. Further, the homogenates were undergone centrifugation (12,000 *g* for 15 min) for about 15 min and the resulting supernatants attained collected. The cytokines (TNF-α, IL-1β, and IL-6) were estimated in the supernatant using the respective rat ELISA kits.

### Western Blotting for a Quantifying NF-κB

We employed Bradford method to estimate the total protein in the homogenate of mammary tissue samples (Bradford, 1976). Around 40 μg protein sample with an equal volume of 2 × laemmli buffer was loaded on each track of 10% polyacrylamide gel and separated by SDS-PAGE. Further, the separated proteins were transmitted to polyvinylidene difluoride membranes (0.2 μm) by electrophoresis. After transferring the proteins, the PVDF membranes were blocked in 3% BSA in Tris-buffered saline and 0.2% Tween-20 for 1 h at room temperature and probed with NF-κB antibodies and β-actin rabbit monoclonal antibody overnight at 4°C. The blots were washed in Tris-buffered saline and 0.2% Tween-20 and then kept with anti-rabbit HRP-labeled secondary antibody for 1 h at room temperature. After, continuous washings, the band of blots were of visualized by using 3,3′,5,5′-Tetramethylbenzidine (TMB) substrate solution. ImageJ software was employed to quantify the band strengths and data was presented as percentage fold alteration compared with control group.

### Assay for Serum Tumor Markers

Tumor markers alpha-fetoprotein (AFP) and carcinoembryonic antigen (CEA) in serum were quantified based on solid phase ELISA method using UBI MAGIWELL (United States) enzyme immunoassay kit (Macnab et al., 1978).

### Histopathology of Mammary Tumors

Mammary tissues were collected from each animal group and fixed in 10% buffered formalin. Thereafter, the tissues were processed sequentially for dehydration and clearing with acetone and xylene. Further, tissues were impregnated in liquid paraffin for half an hour in hot air oven (56°C) and made into a block. Each block was cut into 5 μm thick slice by rotary microtome and then the slices were transferred into floating bath for removal of wax and then collected on egg albumin coated slide. The slides were incubated in hot air oven at 56°C for about 30 min and cleaned with xylene. Then slides were dipped sequentially in 100, 90, 70, and 50% alcohol for 2 min each. Slides were then kept in running water for 10 min before and after dipping in haematoxylin. Slides were immersed in 1% ammonia solution and again kept under running tap water. Finally, the slides were stained by dipping in 2% eosin solution and washed twice with absolute alcohol. The slide was air dried and then mounted with DPX (mounting media) and covered with glass slip (Bodduluru et al., 2015).

### Statistical Analysis

Statistical analysis was executed by employing GraphPad Prism Version 7. The results were expressed as Mean ± Standard Deviation (SD). One way analysis of variance (ANOVA) followed by Tukey’s test as a *post hoc* test was used to correlate the difference between the variables. Values were considered statistically significant if *p* < 0.05.

## Results

### Effect of Honokiol on Body Weight

**Table 1** shows the effect of alterations on the body weight. We can find a substantial decrease in the body weight of DMBA group (Group III) (*p* < 0.05) compared to vehicle control group (Group I) animals. However, pre-treatment with honokiol (Group IV) significantly prevented lost body weight (*p* < 0.05). However, there was no marked change in body weight in the animals administered with honokiol (Group II) alone when compared to vehicle control animals.

**Table 1:** Effect of honokiol on body weight (g), tumor incidence (%), tumor volume (cm^3^) and tumor weight (g) in experimental group of animals.

Parameters/groups	Control	Control + Honokiol	DMBA	DMBA + Honokiol
Body weight (g)	312.54 ± 10.34	307.83 ± 14.56	212.61 ± 9.23	261.17 ± 11.05
Tumor incidence (%)	0	0	100%	50%
Tumor volume (cm3)	0	0	3.37 ± 0.48#	1.21 ± 0.26*
Tumor weight (g)	0	0	13.75 ± 0.81#	6.53 ± 1.04*


*All data are expressed as mean ± SD for groups of six rats in each. One-way ANOVA followed by Tukey *post hoc* test. Values are statistically significant at *p* < 0.05. ^#^*p* < 0.05 as compared with control animals. ^∗^*p* < 0.05 as compared with DMBA control animals.*

### Effect of Honokiol on Tumor Parameters

**Table 1** presents the effect of DMBA and honokiol on the tumor characteristics. Tumor developed in all the animals of DMBA control group (Group III) (*p* < 0.05); however, tumor development was decreased in the honokiol treated groups (Group IV). Likewise, tumor weight and tumor volume were also reduced (*p* < 0.05) with honokiol treatment (Group IV) when compared to DMBA control group (Group III).

### Effect of Honokiol on Liver Marker Enzymes

Liver marker enzymes act as significant indicators of malignant disorders. The concentrations of liver marker enzymes in the serum, such as transaminases, alkaline phosphatases, and LDH in the experimental groups were shown in **Table 2**. A marked upsurge in the levels of serum marker enzymes was found in DMBA group rats (Group III) (*p* < 0.05) compared to control group (Group I) rats. However, these levels were substantially decreased in the animals treated with honokiol (Group IV) (*p* < 0.05). But, there was no substantial alteration in marker enzymes found in rats treated with only honokiol (Group II) compared to control animals.

**Table 2:** Effect of honokiol on liver marker enzymes in experimental group of animals.

Parameters/groups	Control	Control + Honokiol	DMBA	DMBA + Honokiol
AST	35.57 ± 3.12	33.44 ± 2.86	71.23 ± 3.14#	44.32 ± 3.75*
ALT	20.42 ± 3.06	22.12 ± 1.89	41.33 ± 3.55#	28.42 ± 2.12*
ALP	63.54 ± 3.37	61.28 ± 3.32	118.28 ± 10.58#	92.81 ± 7.44*
ACP	9.57 ± 1.04	10.06 ± 0.92	38.54 ± 2.59#	21.14 ± 1.74*
GGT	6.34 ± 0.57	6.13 ± 0.46	12.39 ± 0.87#	8.81 ± 0.69*
LDH	80.04 ± 5.06	77.56 ± 4.54	134.71 ± 9.32#	95.36 ± 6.22*


*All data are expressed as mean ± SD for groups of six rats in each. One-way ANOVA followed by Tukey *post hoc* test. Values are statistically significant at *p* < 0.05. ^#^*p* < 0.05 as compared with control animals. ^∗^*p* < 0.05 as compared with DMBA control animals. Units: AST and ALT are expressed in mmol of pyruvate liberated/mg protein/min; ALP and ACP are expressed in mmol of *p*-nitrophenol liberated/mg protein/min; GGT is expressed in mmol of *p*-nitroaniline liberated/mg protein/min; and LDH is expressed in mmol of pyruvate liberated/mg protein/min.*

### Effect of Honokiol on Phase I and II Biotransformation Enzymes

**Table 3** presents the levels of Phases I and II metabolizing enzymes in the mammary tissue of experimental group of animals. The levels of Phase I enzymes (Cytochrome P450, Cytochrome b5, EROD and PROD) were observed to be raised markedly in the mammary tissue of tumor induced rats (Group III) (*p* < 0.05) as compared to control (Group I) animals. However, the Phase II enzymes (GST and QR) decreased substantially in the mammary tissue of tumor induced (Group III) rats (*p* < 0.05). On the other hand, the pre-treatment of honokiol (Group IV) to animals significantly reduced the levels of Phase I enzymes and increased the levels of Phase II enzymes (*p* < 0.05). There was no marked alteration in the enzyme levels in honokiol alone (Group II) treated animals when compared to control group animals.

**Table 3:** Effect of honokiol on Phase I and II in experimental group of animals.

Parameters/groups	Control	Control + Honokiol	DMBA	DMBA + Honokiol
Cyt P450	0.42 ± 0.02	0.39 ± 0.03	1.57 ± 0.04#	1.11 ± 0.08*
Cyt b5	0.52 ± 0.04	0.56 ± 0.06	1.45 ± 0.09#	0.94 ± 0.03*
EROD	46.22 ± 3.37	41.88 ± 3.32	20.11 ± 2.53#	32.58 ± 2.47*
PROD	24.66 ± 1.05	23.16 ± 1.32	13.15 ± 1.63#	19.31 ± 1.04*
GST	4.43 ± 0.27	4.13 ± 0.36	3.32 ± 0.47#	5.34 ± 0.35*
QR	0.28 ± 0.01	0.26 ± 0.02	0.17 ± 0.02#	0.35 ± 0.02*


*All data are expressed as mean ± SD for groups of six rats in each. One-way ANOVA followed by Tukey *post hoc* test. Values are statistically significant at *p* < 0.05. ^#^*p* < 0.05 as compared with control animals. ^∗^*p* < 0.05 as compared with DMBA control animals. Units: Cyt P450 and cyt b5 expressed in nmol/mg protein, EROD and PROD expressed in mmol of resorufin formed/min/mg protein, GST expressed in mmol of CDNB conjugated with GSH/min/mg protein, QR expressed in mmol of DCPIP reduced per h/mg protein.*

### Effect of Honokiol on Oxidative Stress Parameters

**Table 4** shows the effect of DMBA and honokiol on antioxidant levels. DMBA challenge substantially raised LPO and nitrite, and decreased the activities of mammary tissue enzymatic and non-enzymatic antioxidants (Group III). Honokiol treatment (Group IV) showed detectable ameliorating effect against DMBA-induced increments of LPO and nitrite levels and refurbished the levels of both enzymatic (SOD and catalase) and non-enzymatic antioxidants (GSH, vitamin C and vitamin E) (*p* < 0.05) to near normal levels. No substantial alteration was found in control group and honokiol alone (Group II) treated group of rats.

**Table 4:** Effect of honokiol on oxidative stress parameters in experimental group of animals.

Parameters/groups	Control	Control + Honokiol	DMBA	DMBA + Honokiol
MDA	0.51 ± 0.08	0.56 ± 0.04	1.43 ± 0.15#	0.89 ± 0.08*
SOD	6.65 ± 0.51	6.77 ± 0.72	14.22 ± 1.16#	8.94 ± 0.71*
CAT	66.41 ± 5.17	64.85 ± 3.22	29.51 ± 3.23#	46.82 ± 3.57*
Nitrite	4.70 ± 0.42	4.95 ± 0.36	11.14 ± 1.24#	7.54 ± 0.57*
GSH	34.16 ± 2.20	33.56 ± 2.52	18.42 ± 0.89#	27.65 ± 2.08*
Vit-C	1.24 ± 0.06	1.27 ± 0.05	0.61 ± 0.04#	0.94 ± 0.05*
Vit-E	1.48 ± 0.07	1.51 ± 0.08	0.65 ± 0.06#	1.17 ± 0.08*


*All data are expressed as mean ± SD for groups of six rats in each. One-way ANOVA followed by Tukey *post hoc* test. Values are statistically significant at *p* < 0.05. ^#^*p* < 0.05 as compared with control animals. ^∗^*p* < 0.05 as compared with DMBA control animals. Units: MDA- nmol/mg of protien, SOD- units/mg protein; Catalase- nanomoles of H_2_O_2_ consumed/min/mg protein; protein; NO-nmol/mg of protien; GSH-μg/mg protein; Vit C- μg/mg protein; Vit E- μg/mg protein.*

### Effect of Honokiol on Serum Tumor Marker Parameters

**FIGURE 3:**
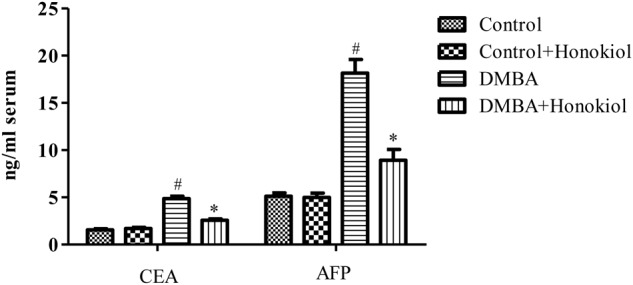
Effect of honokiol on serum tumor markers CEA and AFP in experimental group of animals. All the values are expressed as mean ± SD (*n* = 6). Statistical significance was determined by one-way ANOVA followed by Tukey *post hoc* test; where #*p* < 0.05 when compared with vehicle control group, ^∗^*p* < 0.05 when compared with DMBA control group.

**Figure 3** depicts the effect of honokiol on the concentrations of serum tumor markers CEA and AFP in experimental animals. Tumor induced rats (Group III) showed a marked increase in the levels of these markers as compared to control animals (Group I) (*p* < 0.05). However, honokiol treatment (Group IV) reduced these levels markedly (*p* < 0.05). It confirms the chemopreventive potential of honokiol.

### Effect of Honokiol on Proinflammatory Cytokine Levels

**FIGURE 4:**
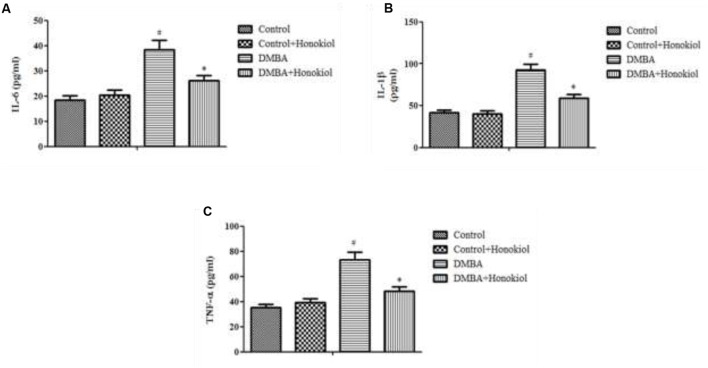
Effect of honokiol on proinflammatory cytokines (IL-6 **(A)**, IL-1β **(B)**, and TNF-α **(C)**). All the values are expressed as mean ± SD (*n* = 6). Statistical significance was determined by one-way ANOVA followed by Tukey *post hoc* test; where ^#^*p* < 0.05 when compared with vehicle control group, ^∗^*p* < 0.05 when compared with DMBA control group.

**Figure 4** presents the effect of honokiol on inflammatory cytokines. TNF-α is a critical inflammatory cytokine liable for tissue inflammation, IL-6 is a pleiotropic cytokine encompasses a role in cell growth and differentiation. Likewise, IL-1β is also a pro-inflammatory cytokine, which has a key role in inflammation. The pro-inflammatory cytokine levels (TNF-α, IL-1β, and IL-6) were increased in DMBA control rats (Group III). However, upon treatment with honokiol (Group IV), mammary cancer bearing animals showed significant (*p* < 0.05) reversal in inflammatory cytokines levels of TNF-α, IL-1β, and IL-6.

### Western Blotting Analysis of NF-κB

**FIGURE 5:**
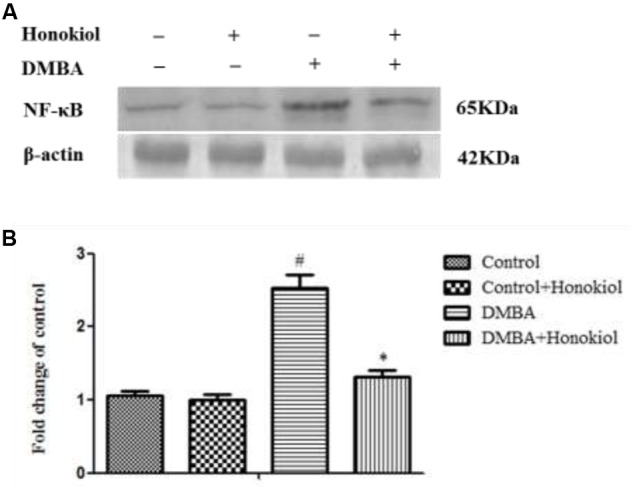
Effect of honokiol on protein expression by western blotting of NF-κB in the experimental group of animals. **(A)** Lanes 1–4 correspond to the breast tissue lysate of groups I–IV, respectively. β-actin was used as loading control of the sample. **(B)** Quantitative data expressing the corresponding protein levels was assessed using ImageJ software and is expressed as percentage fold change compared with loading control. All the values were represented as mean ± SD (*n* = 3). Statistical significance was determined by one-way ANOVA followed by Tukey *post hoc* test; where ^#^*p* < 0.05 when compared with vehicle control group, ^∗^*p* < 0.05 when compared with DMBA control group.

**Figure 5** shows the effect of honokiol on NF-κB expression pattern. NF-κB is a critical transcription factor accountable for tissue inflammation. As compared to control group (Group I), DMBA-treated animals (Group III) demonstrated a significant (*p* < 0.05) increase in the expression of NF-κB levels. Honokiol pre-treatment (Group IV) produced a significant reduction in NF-κB level. No substantial variance was observed in vehicle control (Group I) and honokiol control (Group II) groups.

### Effect of Honokiol on Mammary Tumor Histology

**FIGURE 6:**
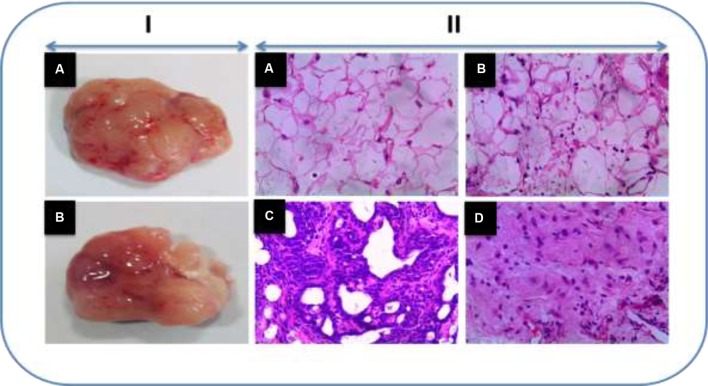
(I) Representative tumor images. (II) The histopathological analysis of sections of breast tissue viewed under light microscope in the experimental animals using H and E staining (H&E). **(A)** Vehicle control animals showing normal architecture. **(B)** Honokiol drug control animals showing normal architecture as that of control animals. **(C)** DMBA treated animals showing demonstrated proliferative lesions with lobular alveolar hyperplasia and mitosis pericentral necrosis. **(D)** Honokiol pre-treated cancer bearing animals showed decreased lobular alveolar damage with near normal architecture.

**Figure 6** shows the histopathological examination of rat mammary tissue sections of experimental groups. Histological sections of cancer induced animals (Group III) demonstrated proliferative lesions with lobular alveolar hyperplasia. On the other hand, mitosis pericentral necrosis, pleomorphism was observed in the group treated with DMBA (Group III). Animals pre-treated with honokiol (Group IV) showed decreased lobular alveolar damage with near normal architecture. No histopathological alterations were seen in the control and honokiol control (Group II) groups.

## Discussion and Conclusion

Honokiol, a traditional Chinese herbal constituent, has been examined in various preclinical studies for its anticancer potential (Fried and Arbiser, 2009). In the present study, we evaluated the preventive effect of honokiol against DMBA-induced mammary cancer in SD rats. In our study, we found that honokiol pre-treatment substantially demonstrated ameliorating effect against DMBA-induced mammary cancer. In the present study, honokiol treatment was started 2 weeks prior to the DMBA administration and continued to till the end of the study period. Chemopreventive potential of several natural products were established using similar model and was the basis for present study (Sahin et al., 2011; Bishayee et al., 2013; Mandal and Bishayee, 2015) Among the several animal models of examining promiscuous nature of breast cancer, we chose DMBA model (Barros et al., 2004). The DMBA-induced mammary cancer in rats is one of the standard preclinical animal models for studying chemopreventive drug development against breast cancer. Incidentally, the DMBA-induced rat mammary cancer model was employed in the development of breast cancer chemo-preventive drug tamoxifen (Jordan and Morrow, 1999). The DMBA-evoked mammary cancer model is relevant to human cancer particularly with regard to origin; in both the cases, experimental as well as human’s cancer arise from ductal epithelial cells (Russo et al., 1990). Furthermore, the DMBA-induced mammary cancer resembles histogenesis, morphological and biochemical features and development of hyperplastic premalignant and malignant lesions that are similar to those of human breast cancer (Witty et al., 1995; Costa et al., 2002).

Several anomalies in physiological processes result in weight loss during cancerous condition (Stein, 1978). In the present study, although there was no marked alteration in the water and food consumption of animals, there was a noticeable reduction in body weight of cancer-induced rats. These findings are in line with a previous study (Cheng et al., 2003). In contrary, in the honokiol pre-treated animals the body weights were found to be near normal. Similar observations were witnessed in an earlier study (Lakshmi and Subramanian, 2014).

Biomarkers are extensively used to monitor cancerous conditions especially to assess the treatment response to the pharmacotherapy. During the carcinogens encounter to the cells, the structural integrity of the cells found to be damaged and eventual cytoplasmic leakage of enzymes into blood stream. AST and ALT are the enzymes related with the formation of amino acids to keto-acids. AST and ALT are pathophysiological diagnostic markers used to gauge tissue damage. The upsurge in the functional activities of AST, ALT ALP, ACP, GGT, and LDH could be mainly because of the leakage from liver cytosol into the blood as a consequence of tissue injury. The tissue injury is a characteristic feature in cancerous atmosphere. The present study observed an increase in the liver enzymes such as AST and ALT in mammary cancer condition; it implies tumor growth. The increased serum ALP concentrations observed in the study may be due to the toxic consequences of DMBA in the liver. GGT, a vital enzyme in glutathione bio-transformation which delivers high intracellular levels of GSH required for conjugation by glutathione *S*-transferase, is associated in protecting cells against toxins and carcinogens. Previous studies demonstrated that there is a greater risk of breast cancer in individuals with raised levels of serum g-GT (Fentiman and Allen, 2010). LDH is acknowledged as a potential marker enzyme for examining the progression of the proliferating malignant cells. Several studies reported the increased levels of LDH in various types of cancers. The increased LDH levels can also be due to the high glycolysis in the cancerous state, which is the only energy-generating way for the unregulated division of malignant cells (Lakshmi and Subramanian, 2014). The raised LDH function in cancer-induced rats can be ascribed to high production of enzymes by proliferated cells and further release of their isoenzymes from injured cells thus making it a sensitive marker for solid neoplasm. In our study, the raised levels of AST, ALT ALP, ACP, GGT, and LDH found in cancer-induced rats were normalized with the pre-treatment of honokiol implicating the safety and non-toxic nature of honokiol.

Normal physiological conditions combat the carcinogens with the assistance of carcinogen-metabolizing enzymes such as Phases I and II metabolizing enzymes. These enzymes function collectively metabolize any xenobiotic that comes in exposure with the body. In the present study, in cancer-induced rats Phase I enzyme levels increased and Phase II enzyme levels decreased. However, honokiol pre-treatment markedly reversed these changes in the enzyme levels.

Several antioxidants present in each human cell prevent the toxic and deleterious effects of oxygen-derived products, which are emerged during normal physiological reactions or during oxidative stress (Chandrashekar et al., 2012). Oxidative stress results in over production of ROS, which makes the antioxidant protective mechanisms more vulnerable to stress insults. Among various antioxidants protective substances SOD and CAT are very much vital in fighting the reactive oxygen species-evoked cellular abnormalities. In our study, DMBA-induced mammary carcinoma containing rats exhibited a significant reduction in the functional activities of enzymatic antioxidants SOD and CAT. Conversely, honokiol treatment substantially reversed the effects of DMBA and restored the antioxidant functions. A few earlier preclinical studies witnessed the similar findings are strengthening our observations of the present study (Anbuselvam et al., 2007; Periyasamy et al., 2015).

Nitrites are one of the DMBA precursors and they also constitute a critical risk factor for breast cancer (Druckrey et al., 1967). In our study, during the active mammary cancer inflammation, higher concentrations of nitrite in mammary tissue were observed as compared to vehicle control group (Levine et al., 1998). This might be due to the result of nitric oxide formed by nitric oxide synthase in immunological cells during the adenoma of preneoplastic transformation (Ambs et al., 1998). Honokiol pre-treatment decreased the nitrite concentrations of mammary tissue compared to DMBA control group. Furthermore, in the present study, DMBA-exposure resulted in increased lipid peroxidation products in mammary tissues, which could cause the formation of tumor by acting with DNA to form MDA-DNA adducts, which evoke genetic abnormalities, leading to carcinogenesis (Kim et al., 2000). The normalcy of LPO levels with the treatment of honokiol is very likely by virtue of its anti-lipid peroxidase activity.

Glutathione is a vital cellular reducing agent, which gives protection against free radicals, peroxides and other toxic compounds (Malhotra et al., 2010). The reduced form of GSH keeps the cellular levels of Vit C and Vit E in their functional forms. These vitamins have several important of biological activities such as immune stimulation, scavenging the free radicals and alteration in metabolic activation of carcinogens (Chandrashekar et al., 2012). In the present study, we found a decrease in GSH, Vitamins C and E levels in DMBA control group. The decreased level of these non-enzymatic antioxidants in DMBA-induced animals could be due to an excessive utilization of these antioxidants for neutralizing massive free radicals produced in this condition. The treatment with honokiol commendably restored the depleted non-enzymatic antioxidants, which might be due to its potent free radical scavenging activity. Even the previous studies in this regard ratify our observations. In breast, lung and other cancer indications; antioxidants provided protective effect against abnormalities in GSH, Vitamins C and E (Lakshmi and Subramanian, 2014; Bodduluru et al., 2015). Therefore, honokiol is a promising agent in preventing oxidative stress-induced breast cancer associated anomalies.

Inflammation plays a crucial role in tumor progression. Proinflammatory cytokines and transcription factor NF-κB; are key molecular contributors for a range of indications such as inflammation to cancer. TNF-α is a crucial cytokine in inflammatory responses, moreover, growing evidence also implicate that TNF-α can act as an endogenous tumor promoter (Balkwill, 2002). NF-κB regulates the expression of several important genes implicated in immunity, inflammation, proliferation, and in defense against apoptosis (Chandrashekar et al., 2012). In our study, with the DMBA challenge we found increased TNF-α level and elevated NF- κB protein expression. Honokiol treatment decreased TNF-α level and downregulated NF- κB protein expression in DMBA treated rats to normal level. Additionally, DMBA caused a raise in the levels of IL-1β and IL-6, which are also very important players in inflammation to cancer pathophysiology. However, honokiol treatment reversed the DMBA-induced anomalous raise in the levels of IL-6 and IL-1β. These observations are in line with previous studies (Qiu et al., 2015) and further support the anticancer activity of honokiol (Arora et al., 2012).

Serum tumor markers are proteins released into the blood stream by cancer cells. A number of serum tumor markers have been implicated for breast cancer detection of which Carcinoembryonic antigen (CEA) and Alpha-fetoprotein (AFP) are most extensively employed (Cheung et al., 2000). Carcinoembryonic antigen is an oncofetal glycoprotein, is expressed in normal mucosal cells and overexpressed in adenocarcinoma of the breast (Haagensen et al., 1978). Alpha-fetoprotein is the major protein of fetal serum formed by the yolk sac and the liver during fetal development. It can be useful as a tumor marker for primary malignancies like hepatocellular carcinoma, germ cell tumors and metastatic cancers (Sell et al., 1983). All these serum tumor markers were increased in the cancer induced rats, whereas honokiol pre-treatment brought these markers to near normal levels. In addition to these biochemical findings, histopathological observations were in correlation with biochemical parameters that further support the cancer preventive effect of honokiol.

Honokiol, an important active component of Chinese herb, Magnolia species; has been found to produce anticipatory promising effects in several types of cancer ailments (Arora et al., 2012). Several phytochemicals, including honokiol, show their cytotoxicity selectively to the anomalous cancer cells and not on the normal healthy cells (Zubair et al., 2017). For instance, one *ex vivo* study demonstrated that honokiol exhibits selective dose dependent cytotoxicity toward primary B-Cell Chronic Lymphocytic Leukaemia (B-CLL) cells, where B-CLL cells were more susceptible to the cytotoxic effects of honokiol compared with normal hematopoietic cells (Battle et al., 2005). Moreover, its safety has been corroborated in several preclinical studies (Arora et al., 2012). Although, clinical studies are still warranted from a broader perspective, alike the other phytochemicals we can anticipate safety and selective cytotoxicity of honokiol clinically too (Zubair et al., 2017).

**FIGURE 7:**
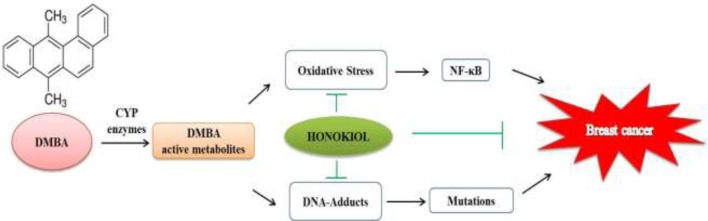
Schematic of probable molecular mechanism of action of honokiol during DMBA induced mammary cancer.

In conclusion, our observations from the present study indicate that honokiol possesses strong potential for preventive activity against DMBA-induced mammary cancer (**Figure 7**). In this study, we attempted in a holistic way to unravel the primal role of honokiol in regulation of various molecular targets of cancer in preclinical trials. Thus, honokiol may definitely direct us to a prospective future of prevention as well as an alternative therapy of breast cancer.

## Author Contributions

ZW was involved in the project design, carried out most of the experiments, and XZ drafted the manuscript. ZW and XZ participated in the molecular, biochemical, and cell biological work. ZW contributed to the animal experiment and data analysis. ZW and XZ conceived and designed the experiments. All authors read and approved the manuscript finally.

## Conflict of Interest Statement

The authors declare that the research was conducted in the absence of any commercial or financial relationships that could be construed as a potential conflict of interest.
